# A high-efficiency primary-side regulation control strategy for half-bridge LLC resonant converter with wide input voltage range

**DOI:** 10.1038/s41598-023-44882-1

**Published:** 2023-10-19

**Authors:** Mian Dai, Daying Sun, Xinyu Wei, LiHui Cong, Chong Wang

**Affiliations:** School of Electronic and Optical Engineering, Nanjing University of Science and Technology, Nanjing, China

**Keywords:** Electrical and electronic engineering, Engineering, Power distribution

## Abstract

The requirement of the half-bridge LLC resonant converter with a wide input voltage range is becoming higher in photovoltaic applications because of its simple structure and low switching loss. Conventional frequency modulation (FM) requires a wide switching frequency range and a high-quality factor circuit design, leading to reduced efficiency and large component volumes at light loads. To solve the problems, a high-efficiency control strategy using adaptive pulse-width and frequency modulation (APWFM) is proposed. APWFM adjusts the gain by changing the switching frequency and duty cycle simultaneously. When the output power is below the reference value, the switching frequency decreases linearly as the output power decreases, and the duty cycle is simultaneously modulated to achieve constant output voltage, so the switching frequency variation range is smaller than FM. This results in improved light or medium load efficiency in a limited frequency range while keeping a small volume of magnetic components. Also, the proposed control strategy is realized with primary-side regulation (PSR) to eliminate the optocoupler and simplify the control circuit. Experimental results demonstrate a significant improvement in efficiency at medium and light loads compared to FM, and the average efficiency is improved by 5% based on low cost and simple operation.

## Introduction

Half-bridge LLC converter, which realizes the soft-switching technique, is widely used in photovoltaic (PV) applications for its simple structure, small volume, and high efficiency. Since the output voltage range of power supply systems for PV is mostly wide^1^, LLC converters using frequency modulation (FM) require a high-quality factor (*Q* > 0.5) circuit design under a wide input voltage range^2^, leading to large component volume. In addition, the switching frequency of FM varies over a wide range, and the switching frequency at light loads can become very high, resulting in difficulty in achieving zero-voltage-switching (ZVS)^3^ and electromagnetic interference (EMI) issues^4^. The light load efficiency is low due to high switching loss and magnetic core loss. These runs in opposition to the miniaturization and the high-power density requirements of micro-inverters in the direction of technology development^5^, and it is difficult to optimize the design of magnetic components^6^.

To maintain a small volume and increase efficiency, modifying the topology network configuration is an effective method that can adjust the root-mean-square (RMS) value of the resonant tank input voltage or change the input or output impedance of the resonant tank. Scholars represented by^7,8^ add other components such as switches on the secondary or primary side of the LLC converter to achieve topological transformation for different load conditions. However, the loss is increased by the additional components and the circuit will become more expensive and complex. Another method is to change the control strategy. Pulse-width modulation (PWM) which only adjusts the duty cycle can reduce the adjustment range of the switching frequency because the switching frequency is fixed^9^. To increase the efficiency of.

the LLC resonant converter at light loads, scholars represented by^10,11^ suggest using burst mode (BM). However, using PWM or BM, a high-quality factor circuit design is required, and as the switching frequency is fixed and kept high, switching loss and magnetic core loss is still high. Nonetheless, inherent issues of BM with dynamic response remain unavoidable.

To effectively improve the light load efficiency and maintain a small volume of half-bridge LLC converter with a wide input range without and no additional circuit components, a high-efficiency control strategy using adaptive pulse-width and frequency modulation (APWFM) is proposed in this paper. When the output power is less than the reference value, the switching frequency decreases linearly as the output power decreases, and the duty cycle is simultaneously controlled to realize constant output voltage, so the switching frequency range is limited under light load. When the output power is above the reference value, the duty cycle is maintained at 50%, and only the switching frequency is adjusted to ensure that the loop current is low and reduces the MOSFET ON-state loss. The proposed control strategy uses primary-side regulation (PSR) to obtain information on the output voltage. The digital control circuit is simple in structure and requires few components. A 48 V/300W prototype with an input voltage range of 280–500 V is designed and tested to validate the theoretical analysis.

## Designation of the proposed converter

**Figure 1:**
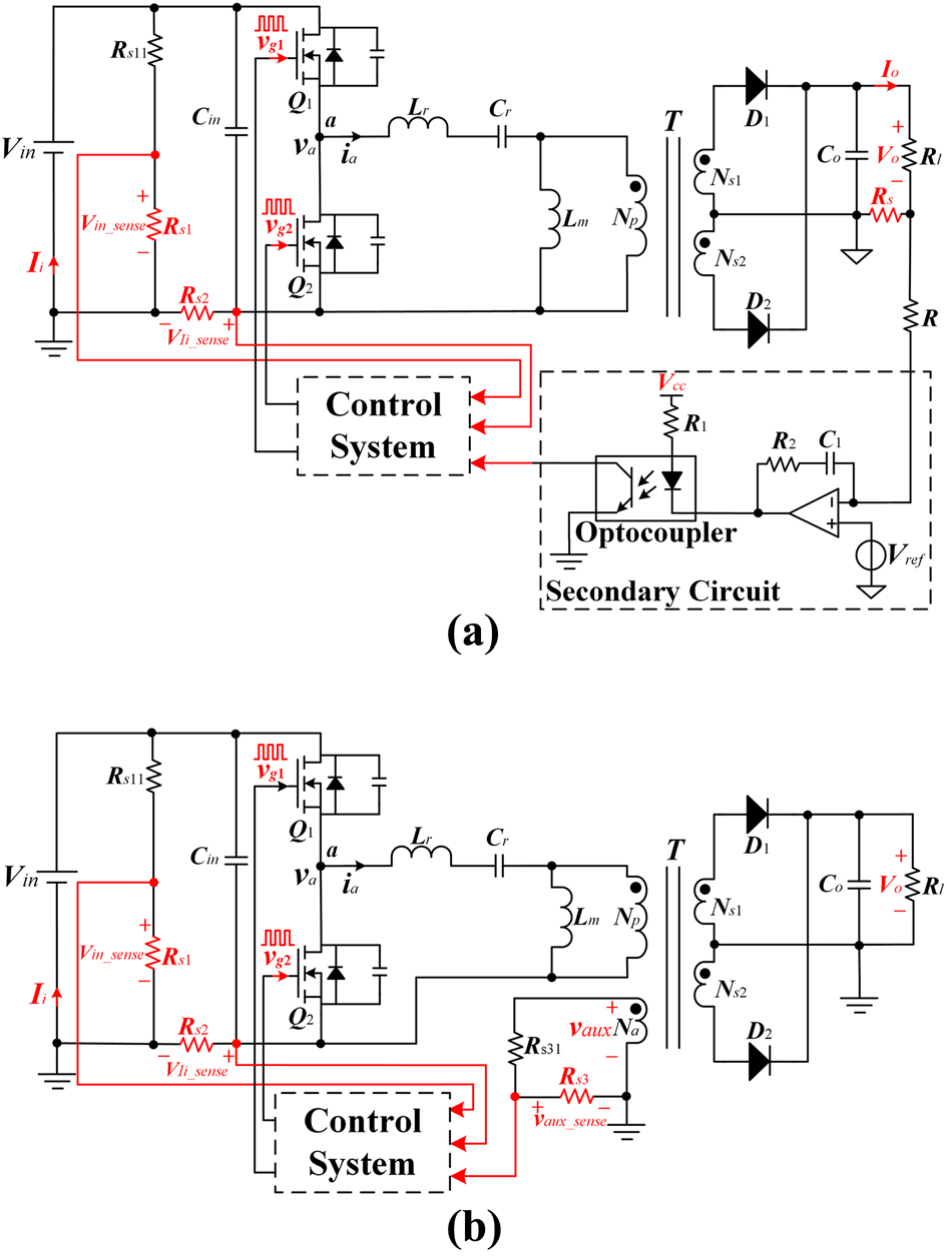
**(a)** Conventional half-bridge LLC resonant converter system. **(b)** Simplified general control system diagram of the proposed control strategy.

A conventional half-bridge LLC resonant converter system using SSF is shown in Fig. 1a. Point *a* is the input node of the resonant tank. However, the nonlinearity, aging, and temperature drift characteristics of the optocoupler result in poor performance and reliability of the converter.

**Figure 2:**
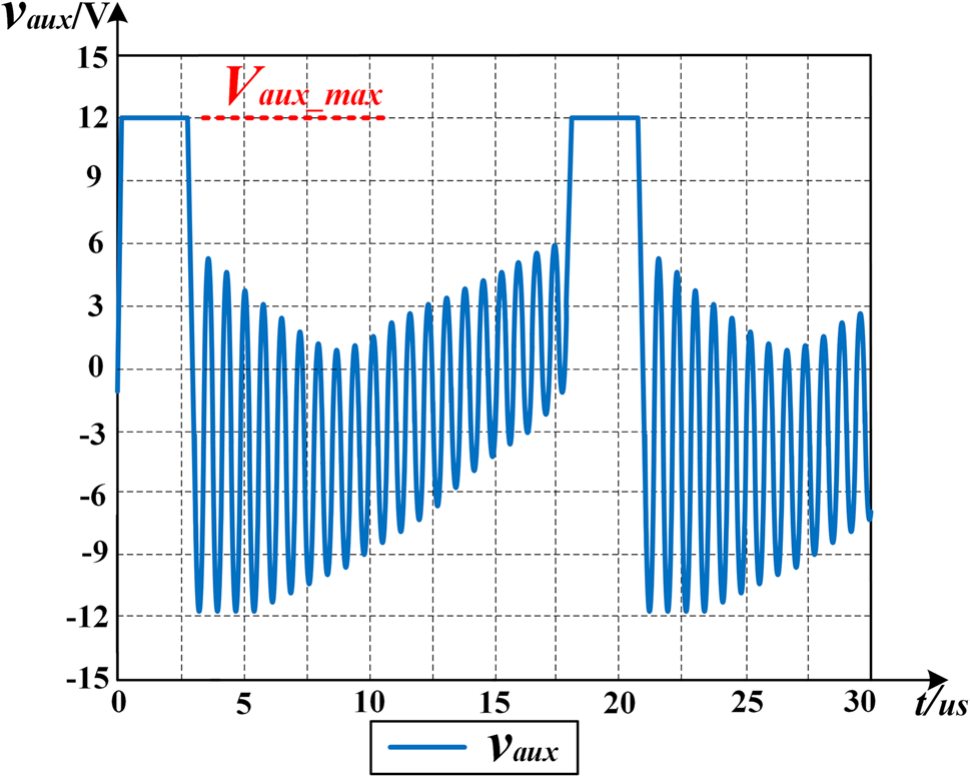
The waveform of *v*_*aux*_ using the proposed control strategy at 10% load at *V*_*in_min*_ = 280 V.

To remove the optocoupler, as shown in Fig. 1b, the PSR is used to collect the information on the output voltage. *v*_*aux*_ is the voltage of the auxiliary winding *N*_*a*_ of *T* and it is an approximate square waveform provided by

1 $$ V_{o} = \frac{{N_{s1} }}{{N_{a} }}V_{aux\_max} = \frac{{N_{s2} }}{{N_{a} }}V_{aux\_max} $$

where *V*_*aux_max*_ is the maximum value of the *v*_*aux*_ as can be seen in Fig. 2.

Using PSR, only the sampling resistor and voltage divider resistor are required. The control system can immediately sample the voltage to estimate the output voltage. Compared to the conventional SSF, the components are removed such as the optocoupler and secondary circuit. PSR significantly reduces the cost of the system, control loss, optimizes the transformer design, and thus, improves efficiency.

## APWFM

### APWFM operational principles

The idea of APWFM is designed as when the load is relatively light, the duty cycle is no longer maintained at 50% but decreased, therefore the range of switching frequency can be reduced; when the load is relatively heavy, the duty cycle is maintained at 50% and only the switching frequency is adjusted to ensure that the loop current is low and reduce the on-state loss. The case when the duty cycle is maintained at 50% is similar to FM, then the following will analyse the case when the duty cycle* D* is below 50%.

**Figure 3:**
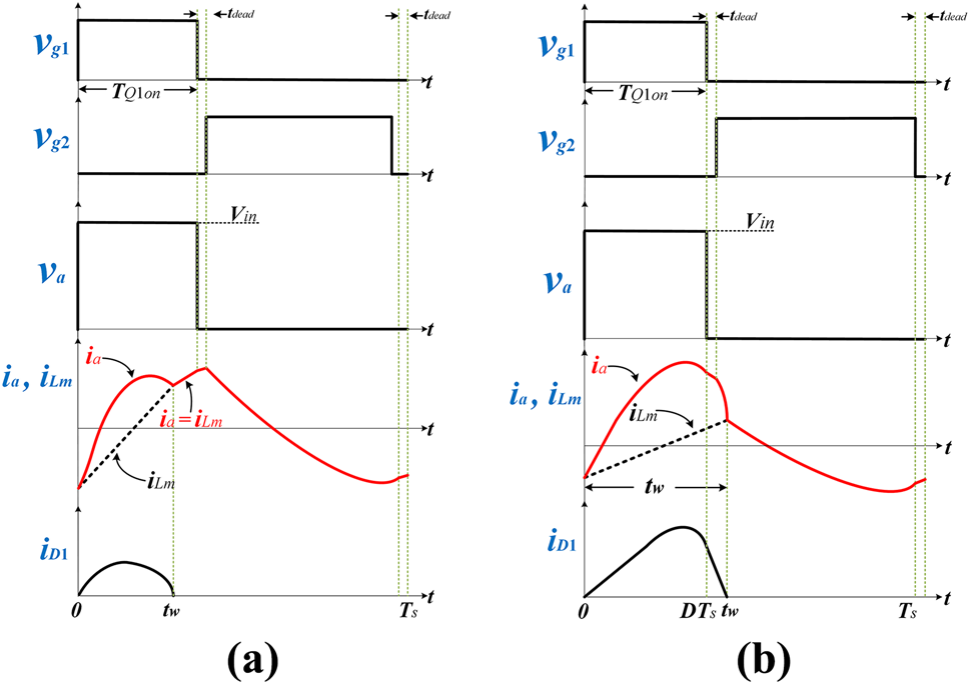
Key waveforms of **(a)** State 1 of APWFM and **(b)** State 2 of APWFM.

To narrow the switching frequency variation range and cater to a wide input range, the duty cycle *D* is modulated below 50% based on the load and input voltage conditions using APWFM. According to the power conduction duration of the secondary rectifier diode *t*_*w*_, APWFM can be divided into two working states. If $0 \le t_{w} < DT_{s}$, the converter enters State 1; If $DT_{s} \le t_{w} \le T_{s} /2$, the converter enters State 2. Figure 3a,b show key waveforms of State 1 and State 2 respectively.

### Gain analysis

**Figure 4:**
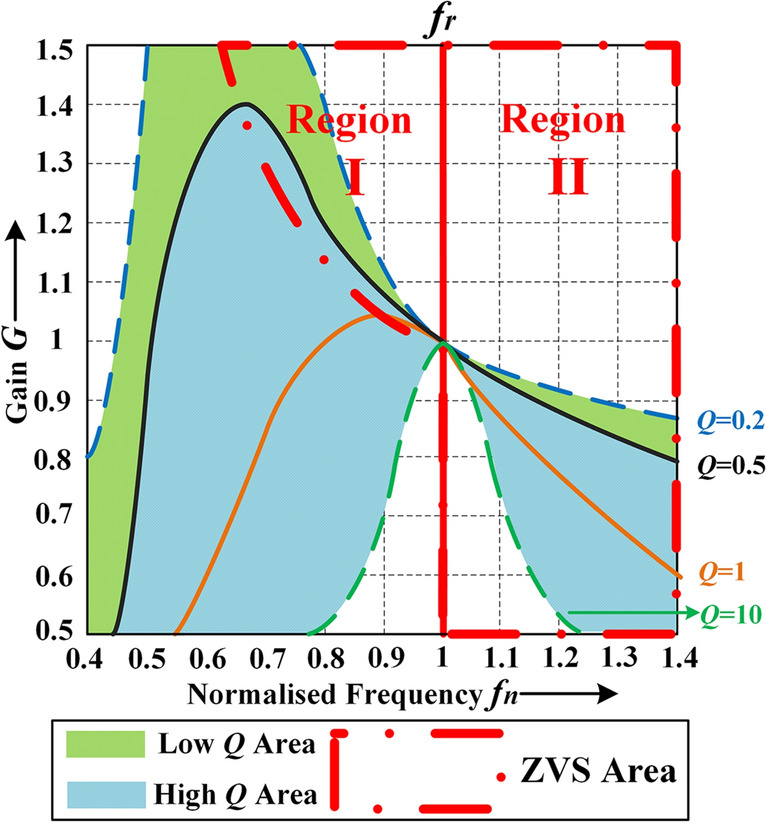
Gain curve using FM.

**Figure 5:**
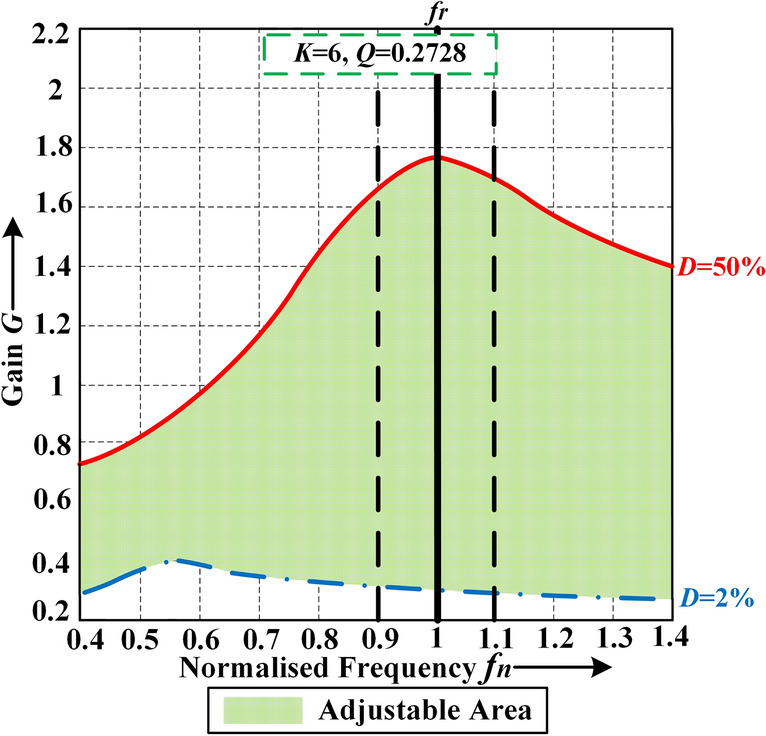
Gain curve using APWFM.

The gain of the resonant tank when using FM can be expressed as (see (2)). As in Fig. 4, if the *Q* value of the converter is not particularly high and the gain can be above 1, the ZVS area is divided into two regions: Region I where the switching frequency is below the resonant frequency, and Region II where the switching frequency is above or equal to the resonant frequency. Regardless of low *Q* or high *Q*, if the range of gain adjustment is large when using FM, a wide switching frequency variation range will be introduced and high efficiency under low gain value cannot be guaranteed especially under light or medium load. The gain of the resonant tank when using APWFM can be expressed as (see (3)). Figure 5 shows the normalized gain curve for a half-bridge LLC converter with *K* = 6 and *Q* = 0.2728 using APWFM at different frequencies. When the duty cycle* D* is 2%, the gain can be reduced to about 0.3. If the normalized frequency *f*_*n*_ is limited to [0.9, 1.1], the minimum gain can also be modulated from 0.3 to 1.65 just by adjustment of *D*. It can be concluded that if the duty cycle is considered, a wide range of gain adjustment is achieved over a switching frequency range smaller than that of FM. It can obtain an additional gain range wider than that using FM, and the gain is not limited by the *Q* value.

### APWFM design

2 $$ G_{dc} = g\left( {f_{n} ,K,Q} \right) = \frac{{K\left( {f_{n} } \right)^{2} }}{{\sqrt {\left\{ {\left( {1 + K} \right)\left( {f_{n} } \right)^{2} - 1} \right\}^{2} + \left\{ {QKf_{n} \left[ {\left( {f_{n} } \right)^{2} - 1} \right]} \right\}^{2} } }} $$

3 $$ G_{prop} = \frac{{2f_{s} C_{r} \left( {1 - \cos \frac{D}{{f_{s} \sqrt {L_{r} C_{r} } }}} \right)}}{{\sqrt {L_{r} C_{r} } \left( {\frac{{f_{s} }}{{n^{2} R_{l} }} + \frac{D}{{2L_{m} }}} \right)\sin \frac{D}{{f_{s} \sqrt {L_{r} C_{r} } }} + \frac{1 - D}{{n^{2} R_{l} }} + \frac{{f_{s} C_{r} \left( {1 - \cos \frac{D}{{f_{s} \sqrt {L_{r} C_{r} } }}} \right)}}{1 - D}}} $$

**Figure 6:**
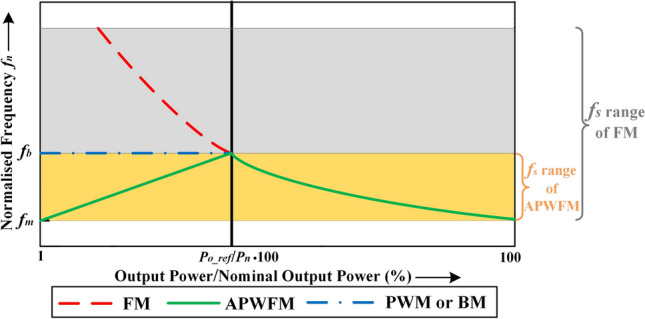
Adjustment curve of switching frequency using APWFM.

According to the idea of APWFM mentioned in section "APWFM Operational Principles", the boundary value needs to be determined whether the duty cycle changes. Figure 6 shows the adjustment curve of switching frequency using APWFM. *P*_*o_ref*_ is the boundary value of the output power *P*_*o*_ for whether the duty cycle starts to decrease from 50%. When *P*_*o*_ is above *P*_*o_ref*_, the duty cycle is fixed at 50%, the switching frequency is regulated in the same way as the ZVS region of the FM in Fig. 4, which guarantees high efficiency at heavy loads. When *P*_*o*_ is below *P*_*o_ref*_, the duty cycle is set to below 50%, and the switching frequency is reduced in a linear law which can be expressed as

4 $$ f_{s} - f_{b} = \frac{{f_{b} - f_{m} }}{{P_{o\_ref} - 1\% \cdot P_{n} }}\left( {P_{o} - P_{o\_ref} } \right) $$

where *P*_*n*_ is the nominal output power, *f*_*b*_ is the switching frequency at the output power of *P*_*o_ref*_ and *f*_*m*_ is the switching frequency at the output power of *P*_*n*_. Therefore, *f*_*b*_ and *f*_*m*_ can be calculated from (see (5)–(8)).

As in Fig. 6, if PWM or BM is used when *P*_*o*_ is below *P*_*o_ref*_, the switching frequency is fixed at *f*_*b*_ which is easy to operate. However, the switching frequency tends to be high when using PWM or BM, which will result in high magnetic loss and turn-OFF loss. If using APWFM, the switching frequency decreases as the output power decreases, therefore the turn-OFF loss and magnetic core loss can be reduced and the switching frequency range is small.

**Figure 7:**
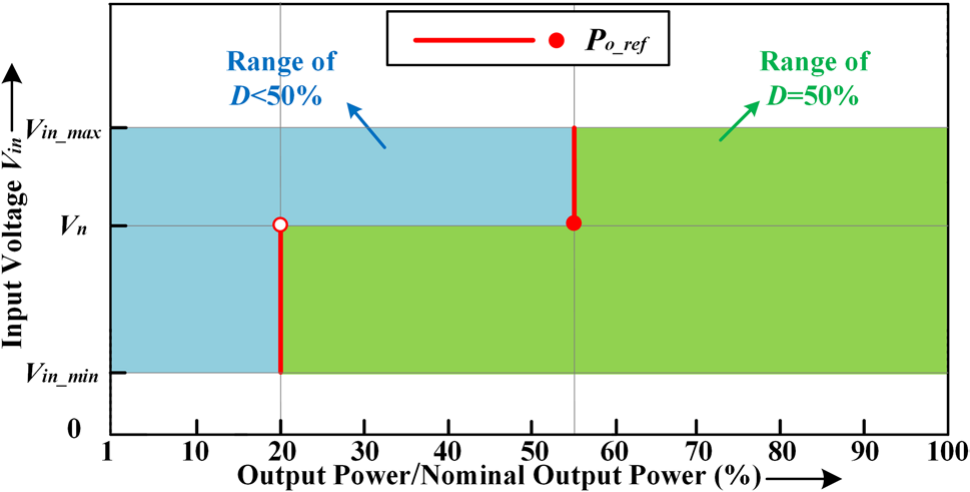
Division diagram for the boundary output power value based on the converter with *Q* = 0.2728 and *K* = *6.*

Based on the power loss model, combined with Figs. 4 and 5, the optimal *P*_*o_ref*_ can be obtained under different load conditions. Figure 7 shows the division diagram for *P*_*o_ref*_ based on the converter with *Q* = 0.2728 and *K* = 6. When the input voltage is below the nominal input voltage *V*_*n*_, *P*_*o_ref*_ is set to 20% of *P*_*n*_. The duty cycle is reduced below 50% when *P*_*o*_ is below *P*_*o_ref*_. When the input voltage is above *V*_*n*_, *P*_*o_ref*_ is set to 55% of *P*_*n*_. The duty cycle is reduced below 50% at medium or light loads when *P*_*o*_ is below *P*_*o_ref*_.

**Figure 8:**
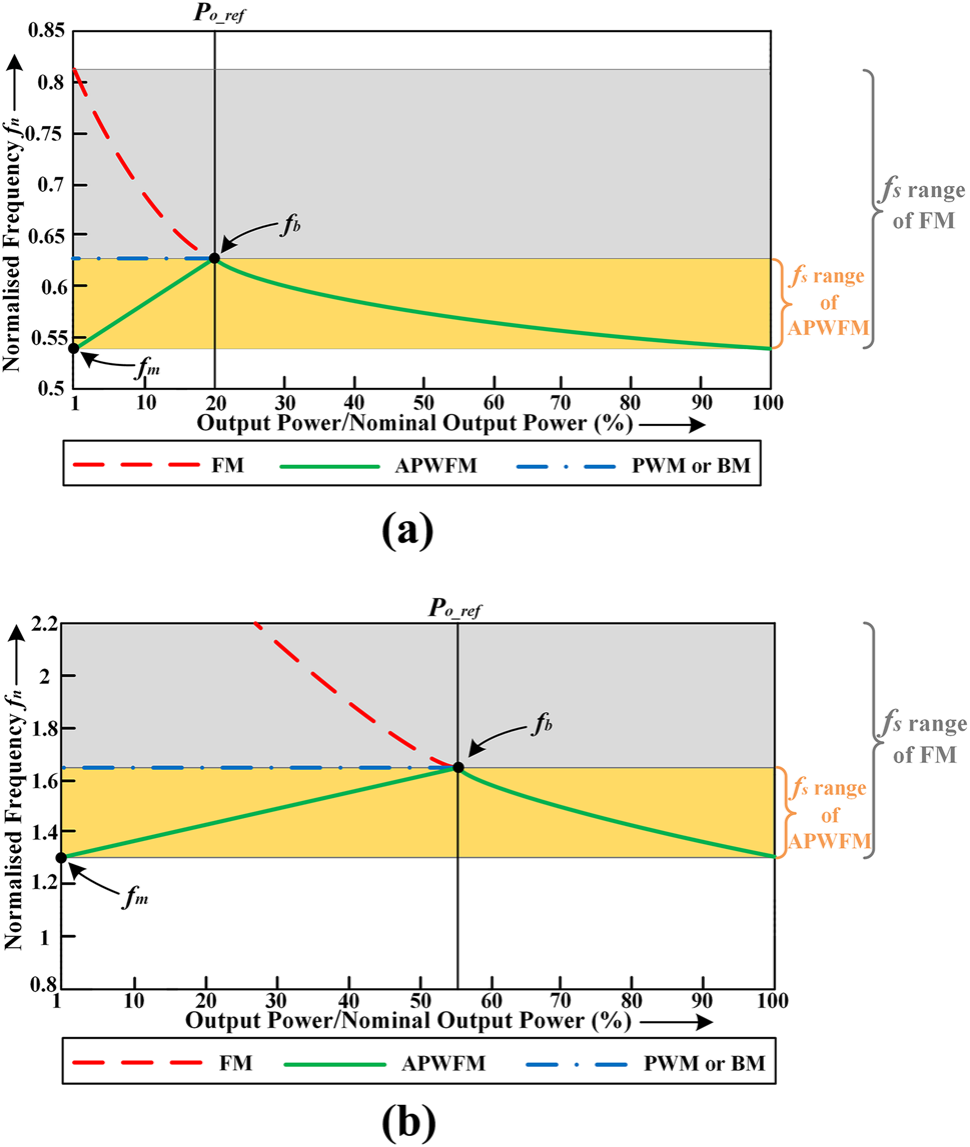
Adjustment curves of switching frequency at, **(a)** *V*_*in_min*_ = 280 V and **(b)** *V*_*in_max*_ = 500 V.

Figure 8a,b show adjustment curves of switching frequency at minimum and maximum input voltage respectively for a 48-V/300-W half-bridge LLC resonant converter with *V*_*n*_ = 400 V and an input voltage range of 280 − 500 V. It can be seen that the switching frequency range with APWFM at *V*_*in_min*_ = 280 V is about 1/3 of that of FM. Also, if using only FM, the switching frequency increase significantly where the maximum value may be around 2.3 MHz at *V*_*in_max*_ = 500 V, which is difficult to operate. However, the switching frequency range with APWFM at *V*_*in_max*_ = 500 V is just 33 kHz. As a result, using APWFM, the switching frequency adjustment range is significantly reduced compared to FM, especially when the required gain is low, the switching frequency using FM increases very high and is difficult to operate, while APWFM allows smooth operation.

After the switching frequency is determined, the duty cycle can be adjusted based on the sampled output voltage information. In this article, the duty cycle is regulated using the PID (Proportional Integral Derivative) algorithm, which is accurate and adaptive. Finally, the optimal combination of switching frequency and duty cycle can be obtained.

## Loss analysis

This chapter will focus on the analysis of the losses of APWFM, as well as a comparative analysis of the losses of FM in the corresponding cases.

### Turn-ON loss

5 $$ G_{dc\_b} = g_{1} \left( {f_{s} } \right) = \frac{{K\left( {f_{s} /f_{r} } \right)^{2} }}{{\sqrt {\left\{ {\left( {1 + K} \right)\left( {f_{s} /f_{r} } \right)^{2} - 1} \right\}^{2} + \left\{ {\frac{{Kf_{s} L_{r} \cdot P_{o\_ref} }}{{4n^{2} V_{o}^{2} }}\left[ {\left( {f_{s} /f_{r} } \right)^{2} - 1} \right]} \right\}^{2} } }} $$

6 $$ G_{dc\_m} = g_{2} \left( {f_{s} } \right) = \frac{{K\left( {f_{s} /f_{r} } \right)^{2} }}{{\sqrt {\left\{ {\left( {1 + K} \right)\left( {f_{s} /f_{r} } \right)^{2} - 1} \right\}^{2} + \left\{ {\frac{{Kf_{s} L_{r} \cdot P_{n} }}{{4n^{2} V_{o}^{2} }}\left[ {\left( {f_{s} /f_{r} } \right)^{2} - 1} \right]} \right\}^{2} } }} $$

7 $$ f_{b} = g_{1}^{ - 1} \left( {G_{dc\_b} } \right) = g_{1}^{ - 1} \left( {\frac{{2nV_{o} }}{{V_{in} }}} \right) $$

8 $$ f_{m} = g_{2}^{ - 1} \left( {G_{dc\_m} } \right) = g_{2}^{ - 1} \left( {\frac{{2nV_{o} }}{{V_{in} }}} \right) $$

Both FM and APWFM operate in the ZVS region in Fig. 4. However, when the switching frequency is adjusted to a very high value using FM, ZVS may be lost, in which case it is recommended to change the control strategy. In this article, the turn-ON loss of FM and APWFM is considered to be 0.

### Turn-OFF loss

**Figure 9:**
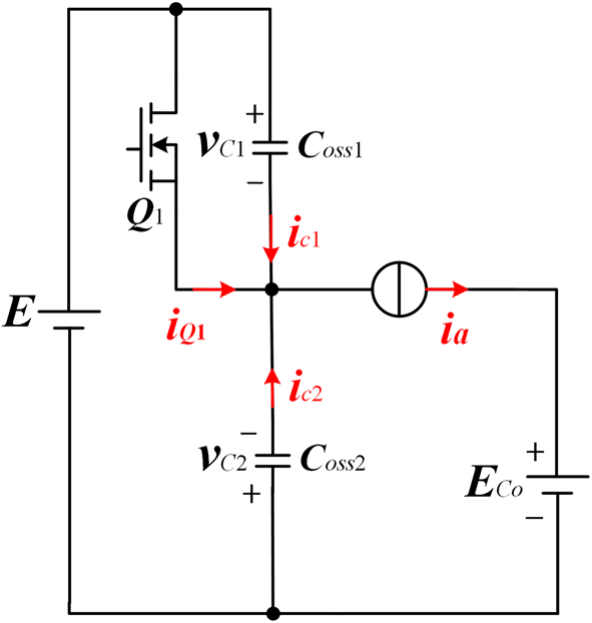
Equivalent circuit for the MOSFET turn-OFF operation.

The equivalent circuit for the MOSFET turn-OFF operation is shown in Fig. 9.

The variation law of the turn-OFF current of *Q*_1_(*Q*_2_) is considered linear. Thus, the current during the process of the *Q*_1_ off is

9 $$ i_{{Q_{1} }} \left( t \right) = I_{off} - \frac{{I_{off} }}{{t_{off} }}t $$

where *I*_*off*_ is the initial value of the current when the MOSFET is turned off, *t*_*off*_ is the time for the current of *Q*_1_(*Q*_2_) to decrease from *I*_*off*_ to 0.

After *Q*_1_ is turned off, the current of the resonant tank keeps negative. Meanwhile, it begins to charge *Q*_1_' s output capacitor *C*_*oss*1_ and discharge *Q*_1_' s output capacitor *C*_*oss*2_. Therefore, the current *i*_*c*1_ flowing through *C*_*oss*1_ and the terminal voltage *i*_*Q*1_ of *Q*_1_ are

10 $$ i_{{C_{1} }} \left( t \right) = i_{{C_{2} }} \left( t \right) = \frac{1}{2}\left( {I_{off} - i_{{Q_{1} }} \left( t \right)} \right) = \frac{{I_{off} }}{{2t_{off} }}t $$

11 $$ v_{{Q_{1} }} \left( t \right) = v_{{C_{1} }} \left( t \right) = \frac{1}{{C_{oss1} }}\mathop \smallint \nolimits_{0}^{{t_{off} }} i_{{C_{1} }} \left( t \right)dt. $$

The equation for the turn-OFF loss is

12 $$ P_{{Q_{1} off}} = \frac{2}{{T_{s} }}\mathop \smallint \nolimits_{0}^{{t_{off} }} i_{Q1} \left( t \right)v_{Q1} \left( t \right)dt. $$

In the region where *f*_*n*_ < 1, *I*_*off*_ can be approximated as

13 $$ I_{off} = \frac{{nV_{o} }}{{4L_{m} f_{r} }}. $$

From the above equations, it can be seen that *I*_*off*_ is a fixed value, therefore the turn-OFF loss increases only with the increase of the switching frequency.

In the region where *f*_*n*_ ≥ 1, *I*_*off*_ can be approximated as

14 $$ I_{off} = \frac{{nV_{o} }}{{4L_{m} f_{s} }} + \frac{{\pi I_{o} \sin (\pi f_{n} )}}{2n}. $$

From the above equations, it can be seen that in the region where *f*_*n*_ ≥ 1, the turn-OFF loss increases rapidly with the increase of the switching frequency because *I*_*off*_ increases as switching frequency increases.

### ON-state loss

To facilitate the analysis, the ON-state loss model is divided into two parts: primary side and secondary side.

The primary-side ON-state loss model includes the MOSFETs ON-state resistance *r*_*Q*1*ds*_(*r*_*Q*2*ds*_), the parasitic resistance of resonant inductor *r*_*Lr*_, the parasitic resistance of resonant capacitor *r*_*Cr*_, and the parasitic resistance of the secondary winding of the transformer *r*_*pri*_. Since PSR is used, the primary-side ON-state loss model also needs to consider the parasitic resistance of the transformer auxiliary winding *r*_*aux*_.

By the electric power equation, the primary-side ON-state loss can be expressed as

15 $$ P_{on\_p} = (r_{{Q_{1ds} }} + r_{{L_{r} }} + r_{{C_{r} }} + r_{T\_p} + r_{aux} )I_{a\_rms}^{2} $$

where *I*_*a_rms*_ is the RMS value of the current of the resonant tank. The value of *I*_*a_rms*_ is directly related to *P*_*on_p*_. Therefore *P*_*on_p*_ decreases as *f*_*s*_ increases, and it is also related to the designed *L*_*m*_ and *n*.

It is worth mentioning that the number of turns of the auxiliary winding is much less than that of the primary and secondary windings. Therefore *r*_*aux*_ is very low. And the power distributed at the same voltage is also very low, therefore the loss at the auxiliary winding is very low.

On the secondary side, it is essential to equate the ON-state loss model of the rectifier diode as the forward voltage *U*_*F*_ in series with the on-state resistance *r*_*F*_, the parasitic resistance of the secondary winding *r*_*sec*_ and the parasitic resistance of the output capacitor *r*_*Co*_.

The secondary-side ON-state loss *P*_*on_s*_ can be given as

16 $$ P_{on\_s} = P_{T\_s} + P_{on\_D} + P_{{C_{o} }} $$

where *P*_*T_s*_ is the loss of secondary winding of the transformer, can be calculated as

17 $$ P_{T\_s} = r_{T\_s} I_{\sec \_rms}^{2} $$

where *I*_*sec_rms*_ is the RMS value of the secondary-side current, which can be expressed as

18 $$ I_{\sec \_rms} = \left\{ \begin{gathered} \frac{{\sqrt 2 \pi I_{o} }}{4}\sqrt {1/f_{n} } ,f_{n} < 1 \hfill \\ \frac{{\sqrt 2 \pi I_{o} }}{4} \, ,f_{n} \ge 1 \hfill \\ \end{gathered} \right. $$

The ON-state loss of the rectifier diodes* P*_*on_D*_ can be calculated as

19 $$ P_{on\_D} = 2r_{F} I_{D\_rms}^{2} + 2U_{F} I_{D\_avg} $$

where *I*_*D_rms*_ is the RMS value of the current flowing through diode *D*_1_ (*D*_2_) and *I*_*D_avg*_ is the average value of the current flowing through *D*_1_ (*D*_2_). *I*_*D_rms*_ and *I*_*D_avg*_ can be expressed by the following equations respectively

20 $$ I_{D\_rms} = \left\{ {\begin{array}{*{20}l} \frac{{\pi I_{o} }}{4}\sqrt {1/f_{n} } ,&\quad  f_{n} < 1 \\ \frac{{\pi I_{o} }}{4} ,&\quad f_{n} \ge 1 \\ \end{array} } \right. $$

21 $$ I_{D\_avg} = \frac{{I_{o} }}{2} $$

The loss of the output capacitor *P*_*Co*_ can be calculated as

22 $$ P_{{C_{o} }} = r_{{C_{o} }} I_{{C_{o} \_rms}}^{2} $$

where *I*_*Co_rms*_ is the RMS value of the current flowing through the output capacitor *C*_*o*_, which can be expressed as

23 $$ I_{{C_{o} \_rms}} = \left\{ \begin{gathered} I_{o} \sqrt {\frac{{\pi^{2} }}{8}f_{n} - 1} ,f_{n} < 1 \hfill \\ I_{o} \sqrt {\frac{{\pi^{2} }}{8} - 1} \, ,f_{n} \ge 1 \hfill \\ \end{gathered} \right. $$

Therefore, as indicated by the above equations, in the region where *f*_*n*_ < 1, *P*_*on_s*_ is below which in the region where *f*_*n*_ $\ge$ 1, and *P*_*on*_*s*_ decreases rapidly as the switching frequency increases.

### Magnetic core loss

The conventional Steinmetz empirical formula can be used to estimate transformer core loss

24 $$ P_{core} = K_{1} {f_{s}}^{\alpha } \Delta B^{\beta } V_{e} $$

where *K*_1_,$\alpha$,$\beta$ are the Steinmetz constants, which depend on the properties of the magnetic material^12^. $\Delta B$ is the transformed variation of the magnetic field strength and *V*_*e*_ is the transformer core volume.

However, only sinusoidal voltages may be used to calculate loss using the conventional Steinmetz empirical formula. According to the analysis in chapter 3, an improved Steinmetz empirical formula is needed because the core voltage of the proposed control strategy is not sinusoidal^13,14.^

25 $$ \begin{aligned} P_{core} & = \frac{{V_{e} }}{{T_{s} }}\int\limits_{0}^{{T_{s} }} {k_{e} } \left| {\frac{dB\left( t \right)}{{dt}}} \right|^{\alpha } \left( {\Delta B} \right)^{\beta - \alpha } dt \\ \, & = K_{1} f_{s} {f_{eq}}^{\alpha - 1} \Delta B^{\beta } V_{e} \\ \end{aligned} $$

where *f*_*eq*_ is the effective switching frequency which can be expressed as

26 $$ f_{eq} = \left\{ \begin{gathered} 8\frac{{f_{r} }}{{\pi^{2} }},f_{n} < 1 \hfill \\ 8\frac{{f_{s} }}{{\pi^{2} }},f_{n} \ge 1 \hfill \\ \end{gathered} \right. $$

It is evident that *P*_*core*_ is positively related to the switching frequency.

### Effects of parasitic parameters

**Figure 10:**
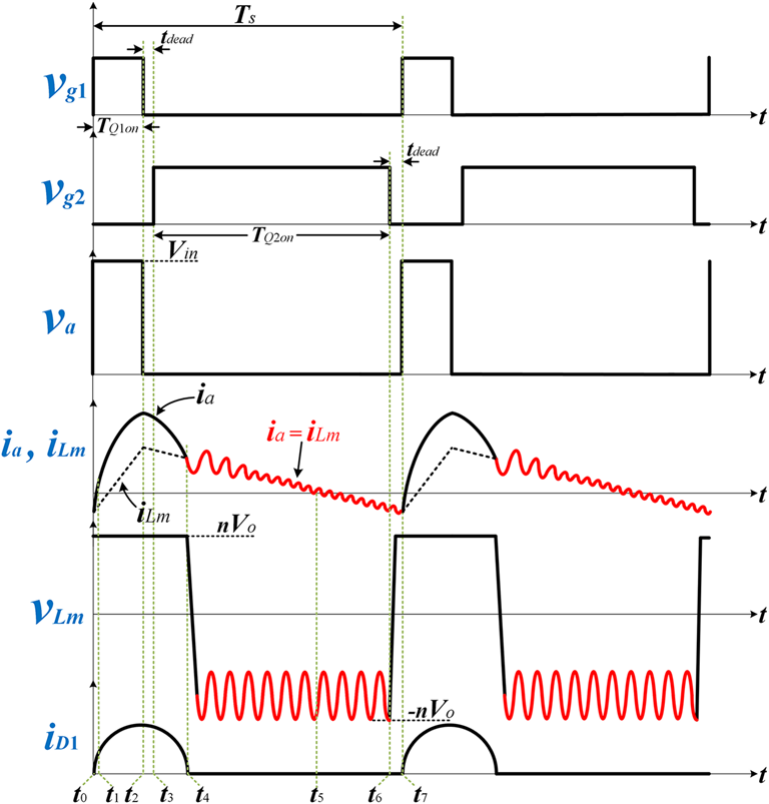
Key waveforms for APWFM considering the parasitic capacitance of the circuit when the duty cycle is below 50%.

The existence of system parasitic capacitance was not taken into account in the previous analysis. To provide isolation against the dc bias, the capacitance of *C*_*r*_ is designed to be large. As shown in Fig. 10, in *t*_4_ ~ *t*_7_, the parasitic capacitors generated by magnetic components and rectifier diodes resonate with *C*_*r*_, *L*_*r*_, and *L*_*m*_ in the resonant tank. This results in a small high-frequency oscillation of the resonant inductor current, accompanied by a small number of zero crossing points which decrease or disappear as the output power increases. Due to the small amplitude of the oscillation and the fact that *Q*_2_ is already on at *t*_5_ and *Q*_1_ is off at this time, the ZVS of *Q*_1_ and *Q*_2_ is unaffected. Therefore, the loss caused by parasitic capacitors is minimal.

### Summary

At ultra-light loads, the conventional method is to use FM to increase the switching frequency significantly which is difficult to operate and causes high loss. In this article, using BM instead of FM to narrow the switching frequency range in that case.

**Figure 11:**
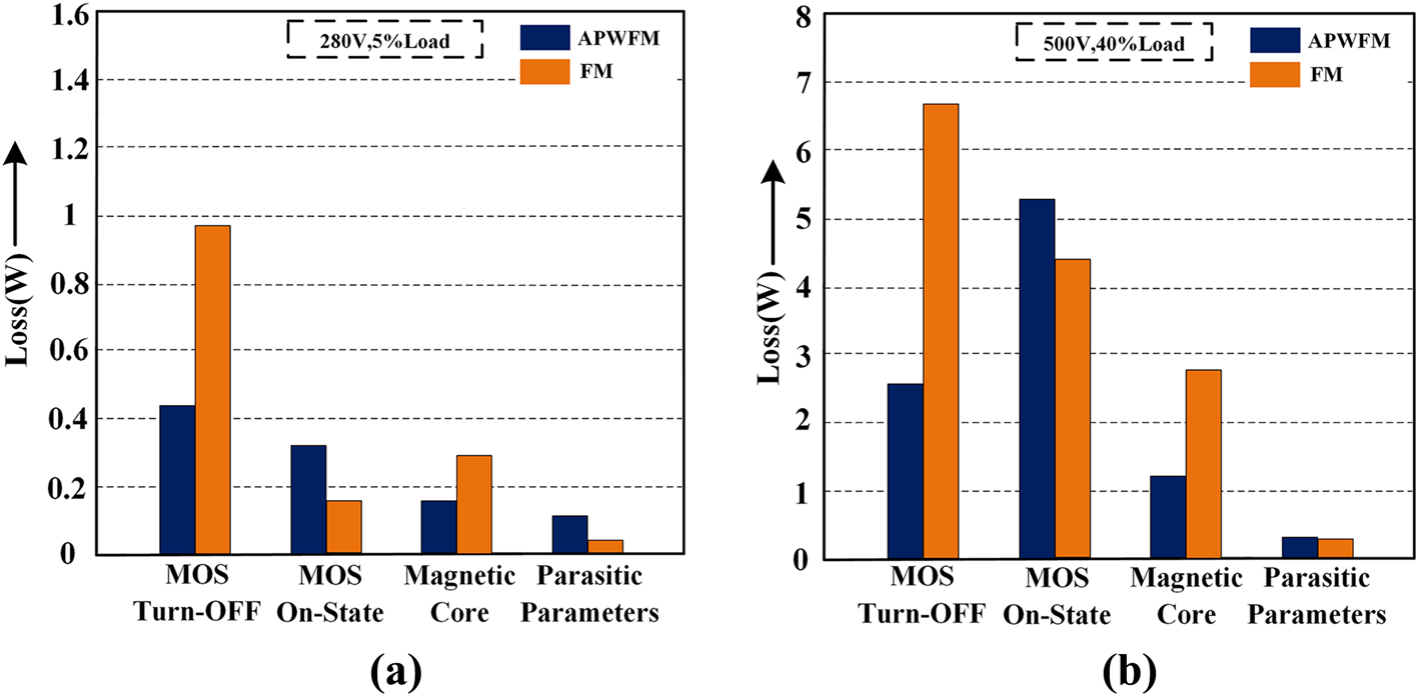
Power loss distribution at. **(a)** *V*_*in_min*_ = 280 V at 5% load using APWFM or FM and, **(b)** *V*_*in_max*_ = 500 V at 40% load using APWFM or FM.

Figure 11a,b show the power loss breakdown of a 48-V/300-W half-bridge LLC resonant converter at *V*_*in_min*_ = 280 V at 5% load and *V*_*in_max*_ = 500 V at 40% load respectively. At light or medium loads, the turn-OFF loss and magnetic core loss are significantly decreased because APWFM reduces the switching frequency compared to FM. The ON-state loss of APWFM is above that of FM, but the difference is manageable. When the output power is above *P*_*o_ref*_, the ON-state loss becomes dominant, therefore the duty cycle needs to be maintained at 50% to minimize the loop current. At the same load operation, especially under light load conditions, the switching frequency of FM is higher than that of APWFM, which causes an increase in current passing through parasitic components. So as shown in Fig. 11b, the parasitic parameter loss difference between APWFM and FM is significantly reduced.

**Figure 12:**
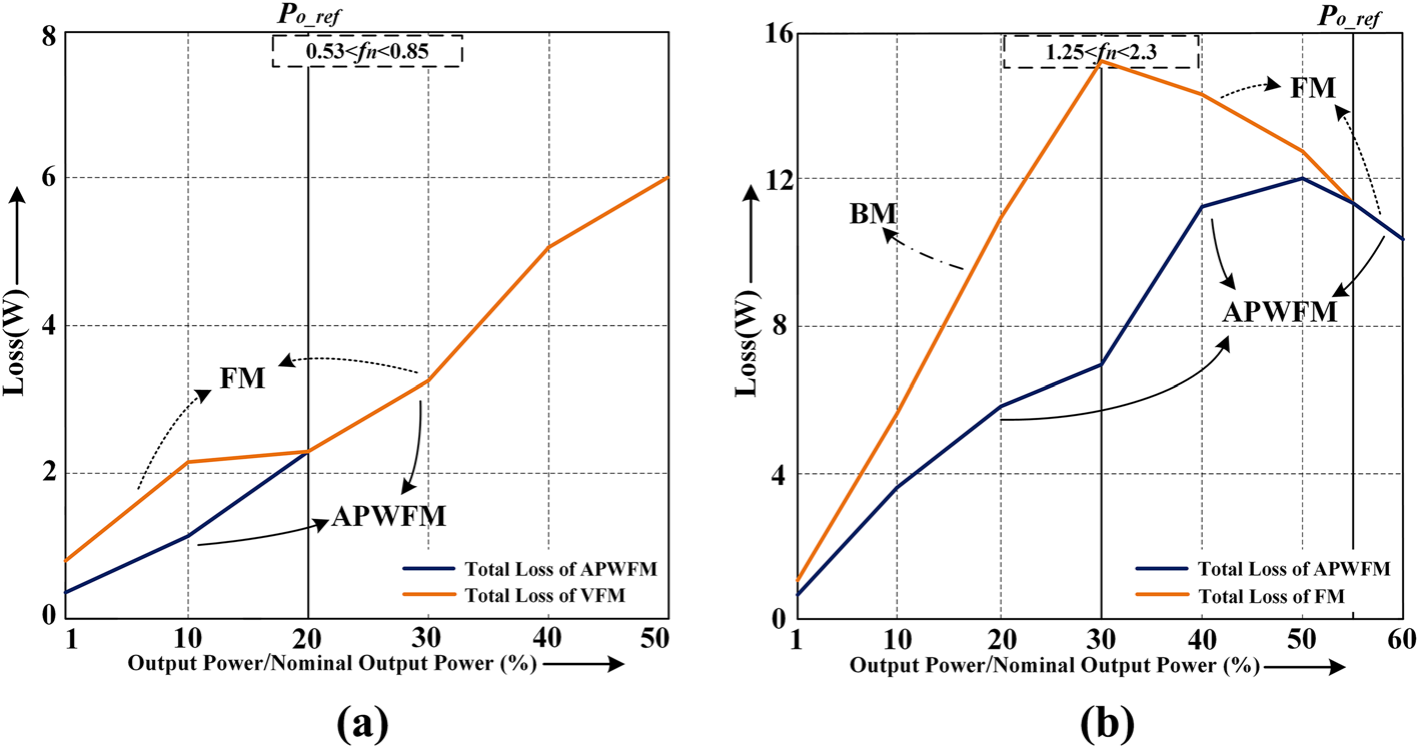
Total loss comparison using APWFM at. **(a)** *V*_*in_min*_ = 280 V using APWFM or FM and, **(b)** *V*_*in_max*_ = 500 V using APWFM or FM + BM.

Figure 12a,b show the total loss comparison of the converter between APWFM and FM or FM + BM at *V*_*in_min*_ = 280 V and *V*_*in_max*_ = 500 V respectively. In conjunction with the above analysis, when the output power is below *P*_*o_ref*_, the total loss of the converter can be reduced if the switching frequency is reduced rather than being fixed or increased as the output power decreases. Compared to FM or BM, the average efficiency of the converter using APWFM is higher.

## Realization of proposed control strategy

### Design of magnetic components

According to the previous analysis, the frequency variation of the proposed control strategy is significantly smaller compared to FM, allowing for the optimization and miniaturization of magnetic components.

Since the proposed control strategy can realize a large range of the gain, it applies to a wide input voltage range, the transformer needs to be designed to allow operation at high frequencies and requires a high flux density. Its specific type can be selected according to the area product (AP) method

27 $$ A_{p} = \left( {\frac{{P_{T} \cdot 10^{4} }}{{2 \cdot \Delta Bf_{s} JK_{u} }}} \right)^{{\frac{1}{1 + X}}} $$

where *P*_*T*_ is the apparent power, *X* is a constant determined by the core used, *K*_*u*_ is the window usage factor and *J* is the current density.

If using the proposed control strategy to control a 48-V/300-W half-bridge LLC resonant converter with an input voltage range of 280–500 V and the resonant frequency equal to 100 kHz, the switching frequency range for the AP method reference is (50 kHz, 163 kHz]. However, if using FM under the same condition, the switching frequency range is (50 kHz, 250 kHz) while BM is recommended when the required gain is very low otherwise the switching frequency will be much high. Under the comparison of the switching frequency range, the feasibility of the design of magnetic components can be seen in combination with Eq. (27).

### Specific control rules

To meet the requirement to minimize the switching frequency variation range, the proposed control strategy compares the product of the input voltage and input current with the input power reference value *P*_*in_ref*_ first. *P*_*in_ref*_ is set to be equal to the input power when the output power is equal to *P*_*o_ref*_.

Since the magnetic loss increases abnormally when the duty cycle is below 2% from Eq. (25), the adjustment range of the duty cycle is (2%, 50%].

**Figure 13:**
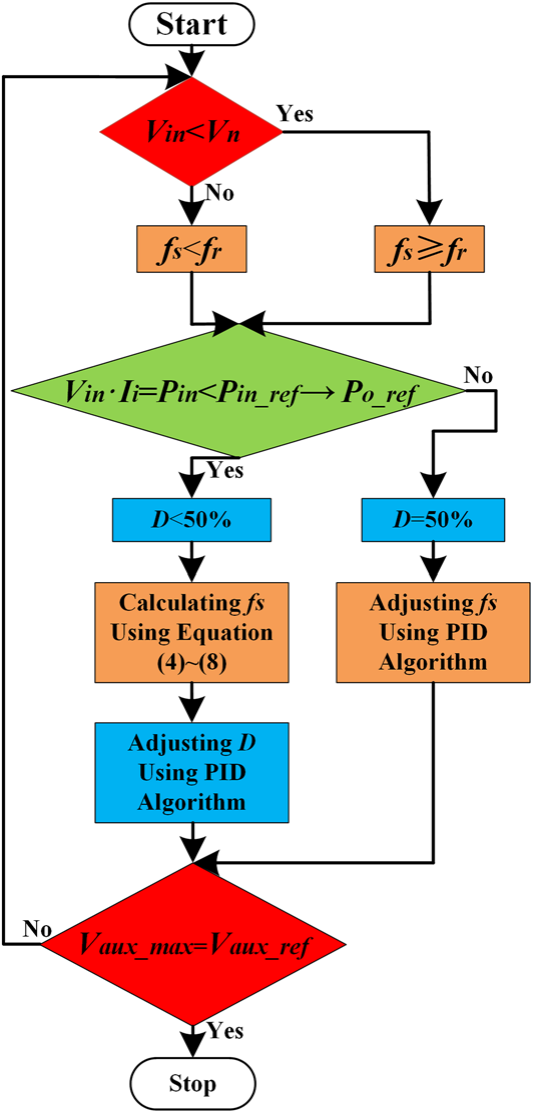
Switching frequency and duty-cycle selection block flowchart.

After acquiring the range of the switching frequency and duty cycle variation, *V*_*aux_max*_ is compared with a reference value *V*_*aux_ref*_. *V*_*aux_ref*_ is set to the maximum value of the *v*_*aux*_ when the converter is at the desired output voltage. If the result is equal, then the desired switching frequency and duty cycle are obtained; otherwise, repeat the process. By ensuring that *V*_*aux_max*_ reaches the reference value *V*_*aux_ref*_ in this way, the desired constant voltage is guaranteed. In Fig. 1b, *V*_*in_sense*_, *V*_*Ii_sense*_, and *V*_*aux_sense*_ are the sampling values of the resistor divider for the input voltage, the input current, and the auxiliary winding voltage respectively. Figure 13 shows the logic flowchart of the Switching frequency and duty-cycle selection block.

## Experimental results

**Figure 14:**
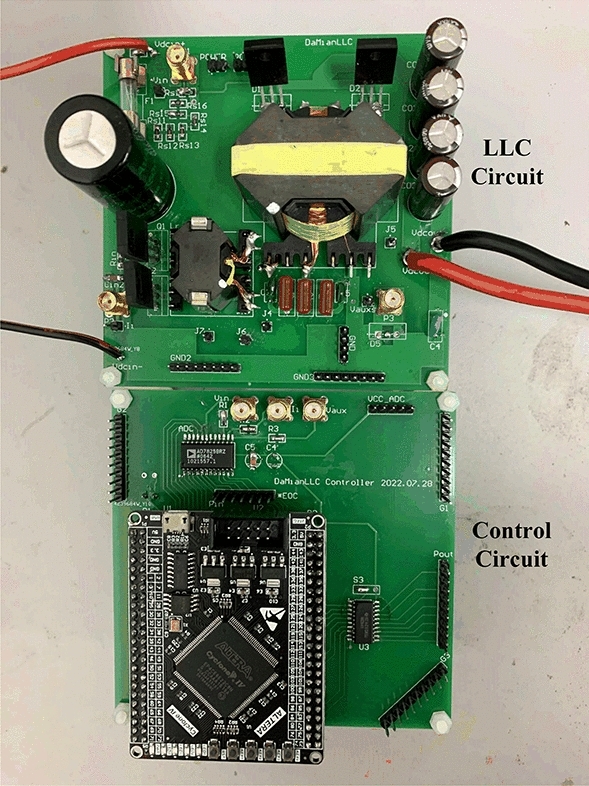
Prototype of the LLC converter.

An experimental prototype of a 48-V/300-W half-bridge LLC resonant converter has been built as shown in Fig. 14, in order to test the functionality and efficiency of the implemented converter are given in Table Table 1. The control system is implemented using FPGA and only one ADC chip, AD7825 from Analog Devices, is used to simultaneously sample the required input voltage, input current, and voltage information of auxiliary winding in three separate sampling cycles, without the need for peripheral circuitry. This way can help to reduce the cost and improve control efficiency.

**Table 1:** Design specifications.

Component	Parameters
Input voltage range (*V*_*in*_)	280–500 V, *V*_*n*_ = 400 V
Output voltage (*V*_*o*_)	48 V
Maximum output Power (*P*_*o_max*_)	300W
Resonant frequency (*f*_*r*_)	100 kHz
Resonant capacitor (*C*_*r*_)	32 nF
Resonant inductor (*L*_*r*_)	48 uH
Transformer (*T*)	Core:RM14, *L*_*m*_ = 470 µH, *N*_*p*_:*N*_*a*_:*N*_*s*1_:*N*_*s*2_ = 31:2:8:8
Output capacitor (*C*_*o*_)	4 $\times$ 100 µF/100 V

According to the analysis in the preceding sections, it is easy to accomplish effective gain regulation at a high *Q* with the LLC resonant converter. Therefore, the experimental prototype circuit used *Q* = 0.2728 (*Q* < 0.5) to test the applicability of the proposed control strategy in the low *Q* situation.

By accomplishing ZVS in the worst situation, ZVS can be guaranteed in all conditions which ensures high efficiency. According to the previous analysis, the ZVS of the low-side MOSFET (*Q*_2_) implies the ZVS of the primary MOSFETs. Therefore, the ZVS performance at the minimum and maximum input voltage when the output power is close to *P*_*o_ref*_ or at very light loads are tested under the proposed control strategy.

**Figure 15:**
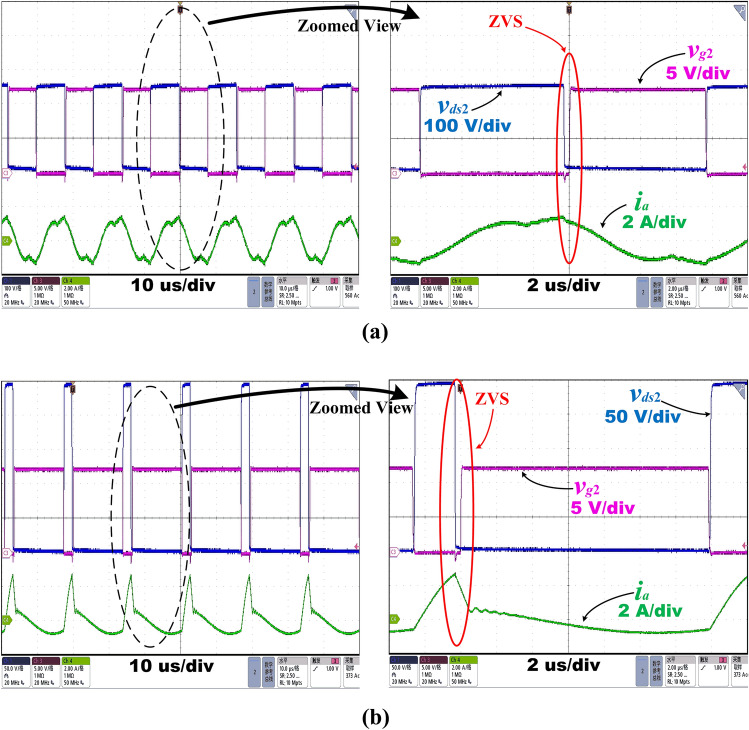
Operating waveforms at *V*_*in_min*_ = 280 V using the proposed control strategy at **(a)**20% load and **(b)**15% load.

Figure 15a shows the MOSFET experimental waveform for the switch *Q*_2_ of drain-source voltage, *v*_*ds*2_, gate-source voltage, *v*_*g*2_, and resonant current, *i*_*a*_, under the proposed control strategy with* D* = 50% and *f*_*s*_ = 62.8 kHz at *V*_*in_min*_ = 280 V at 20% load operation. This is the case when the output power is just equal to the boundary value* P*_*o_ref*_ = 20%*P*_*n*_ at the minimum input voltage. Also, Fig. 15b shows the MOSFET experimental waveform for the switch *Q*_2_ of drain-source voltage, *v*_*ds*2_, gate-source voltage, *v*_*g*2_, and resonant current, *i*_*a*_, under the proposed control strategy with *D* = 12% and *f*_*s*_ = 60.6 kHz at *V*_*in_min*_ = 280 V at 15% load operation. As illustrated in Fig. 15a,b, the ZVS of the primary MOSFETs can be obtained when the switching frequency is in Region I and the output power is close to *P*_*o_ref*_, which proves high efficiency.

**Figure 16:**
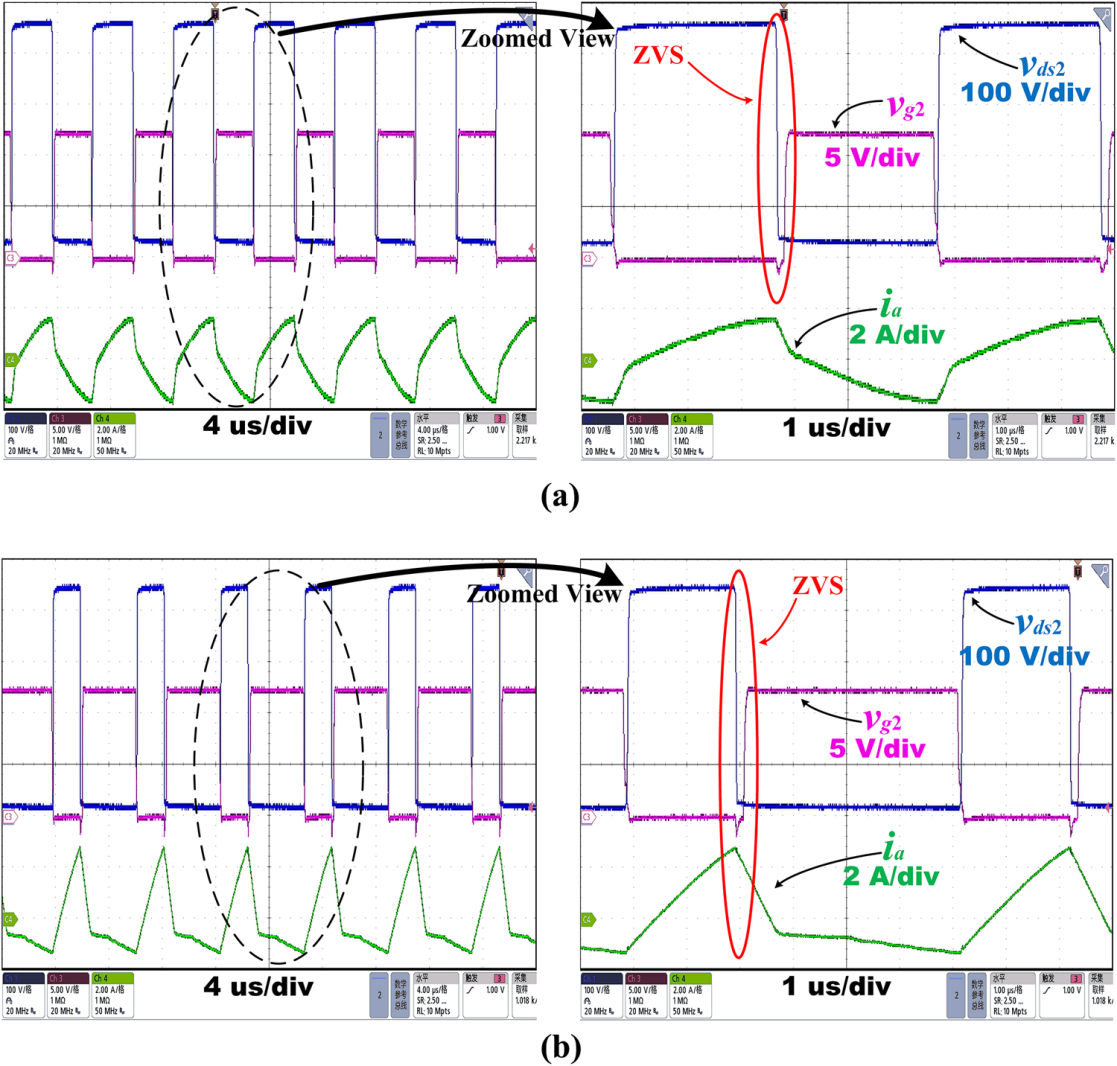
Operating waveforms at *V*_*in_max*_ = 500 V using the proposed control strategy at **(a)**55% load and **(b)**50% load.

Figure 16a,b show the MOSFET drain-source voltage, *v*_*ds*2_, gate-source voltage, *v*_*g*2_, and resonant current, *i*_*a*_, waveform for the switch *Q*_2_ during the converter operation at *V*_*in_max*_ = 500 V at 55% load operation with *D* = 50% and *f*_*s*_ = 163 kHz, and at *V*_*in_max*_ = 500 V at 50% load operation with *D* = 29% and *f*_*s*_ = 160 kHz respectively. These are the cases when the output power is just equal and next to the boundary value *P*_*o_ref*_ = 55%*P*_*n*_ at the maximum input voltage. As can be seen from the figures, when the switching frequency is in Region II, the high efficiency of the converter is still guaranteed at medium loads.

**Figure 17:**
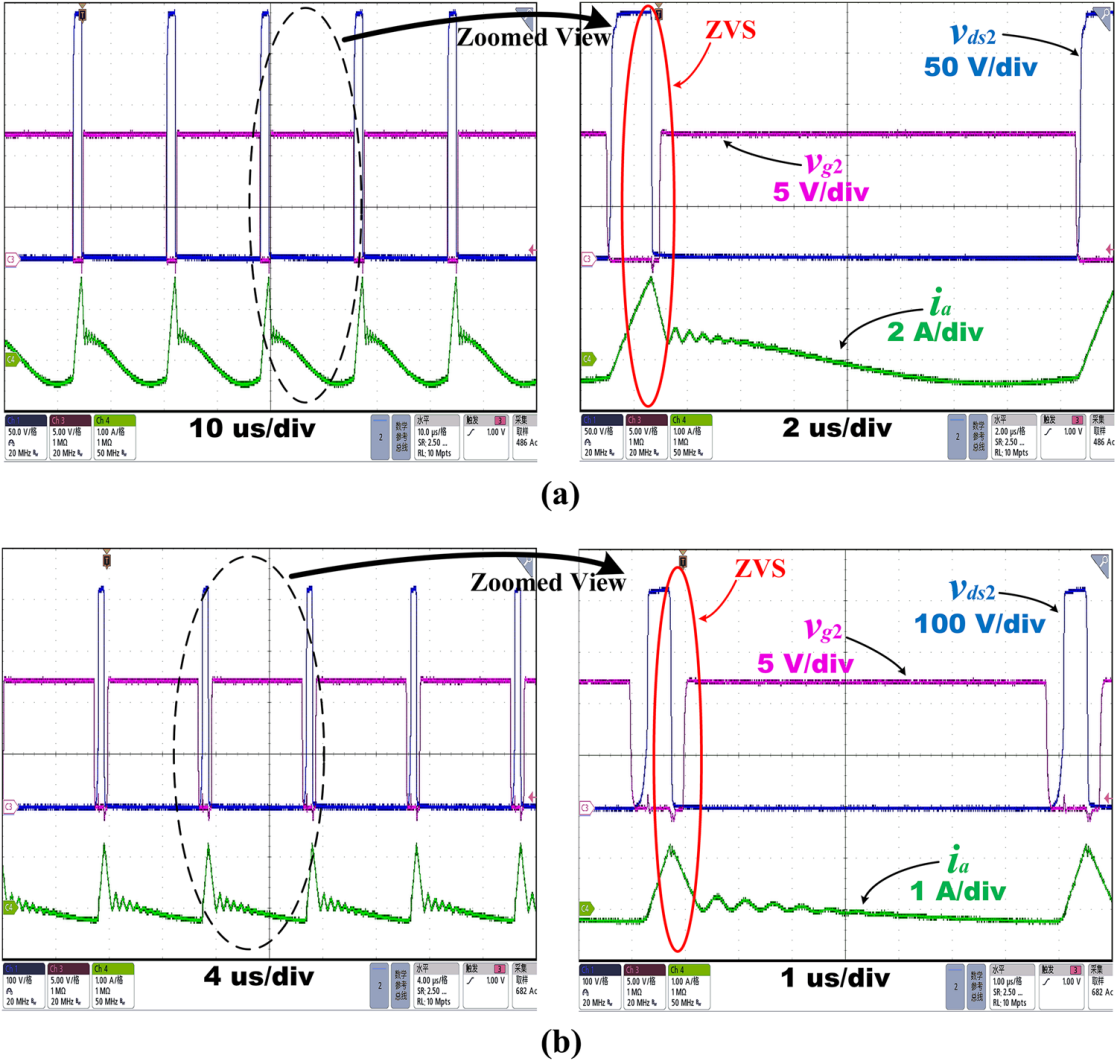
Operating waveforms at 5% load using the proposed control strategy at **(a)** *V*_*in_min*_ = 280 V and **(b)** *V*_*in_max*_ = 500 V.

From the analysis in the above sections, at light loads, FM can cause very high switching frequencies that are difficult to operate or lose ZVS which results in poor efficiency, requiring a change in the control strategy. To validate the analysis of the proposed control strategy under ultra-light loads, Fig. 17a shows the MOSFET experimental waveform for the switch *Q*_2_ of drain-source voltage, *v*_*ds*2_, gate-source voltage, *v*_*g*2_, and resonant current, *i*_*a*_, under the proposed control strategy with *D* = 7.17% and *f*_*s*_ = 56.2 kHz at *V*_*in_min*_ = 280 V at 5% load operation. Using the proposed control strategy, ZVS can be obtained for light loads, and the full load is controlled using only APWFM, without the need to change the control strategy by the time the light load is reached. Figure 17b shows the MOSFET experimental waveform for the switch *Q*_2_ of drain-source voltage, *v*_*ds*2_, gate-source voltage, *v*_*g*2_, and resonant current, *i*_*a*_, under the proposed control strategy with *D* = 4.63% and *f*_*s*_ = 133 kHz at *V*_*in_max*_ = 500 V at 5% load operation. Obtaining ZVS at ultra-light loads proves the guarantee of ZVS at full loads and the guarantee of high average efficiency, validating the previous analysis. Under light load conditions, the small oscillations that occurred in the resonant current in *t*_4_ ~ *t*_7_ according to section "Effects of Parasitic Parameters" result in a small amount of zero-crossing using the proposed control strategy. However, it is experimentally proven that they have a negligible effect on efficiency.

**Figure 18:**
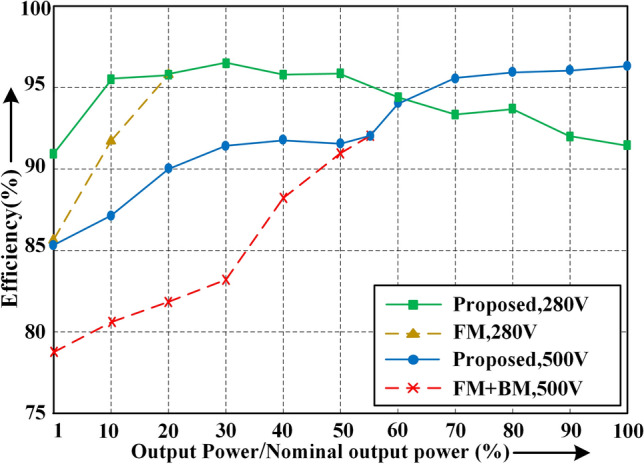
Efficiency chart.

To verify the previous analysis, a specific experimental evaluation of the efficiency of the converter is carried out. It is worth mentioning that when the required gain is very low, similar to the operation in section "Summary", BM is experimentally used instead of FM to compare with the proposed control strategy. Figure 18 demonstrates that the proposed control strategy has an excellent average efficiency under full load conditions with a wide input voltage range. The proposed control strategy significantly improves the efficiency of FM when the duty cycle is below 50%, especially at *V*_*in_max*_ = 500 V at 30% load operation, with an efficiency improvement of 9%.

**Figure 19:**
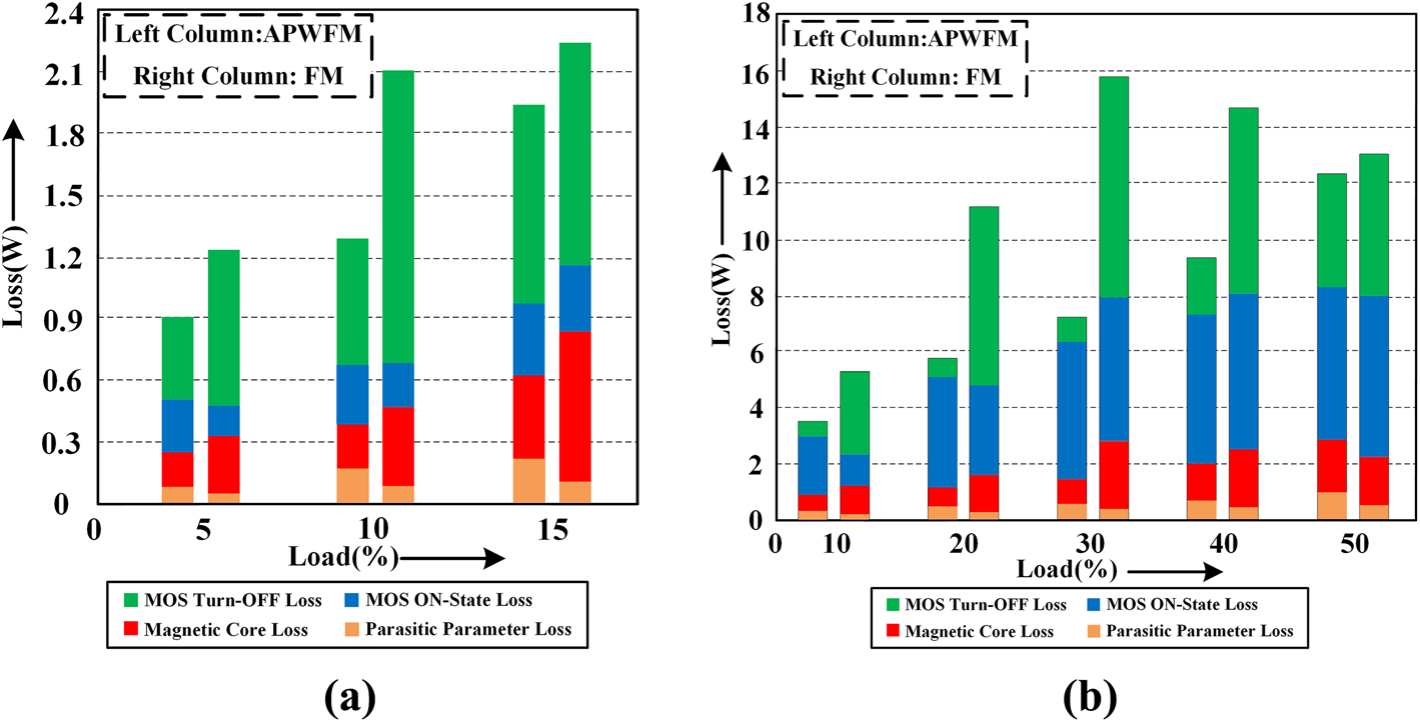
Power loss breakdown **(a)** using the proposed control strategy and FM at *V*_*in_min*_ = 280 V and **(b)** using the proposed control strategy and FM + BM at *V*_*in_max*_ = 500 V.

The experimental power loss breakdown using the proposed control strategy and FM at *V*_*in_min*_ = 280 V is shown in Fig. 19a. With the proposed control strategy in Region I, the turn-OFF loss and magnetic core loss are significantly improved compared to FM because the switching frequency is decreased rather than increased. Parasitic parameter loss increases but is very low in proportion, verifying the previous analysis. Figure 19b shows the improvement in turn-OFF loss in Region II but also demonstrates the dominance of the ON-state loss.

## Conclusion

A control strategy for the half-bridge LLC resonant converter with a wide input voltage range to achieve high efficiency under all load conditions is proposed in this paper. As the load decreases, the switching frequency decreases and the duty cycle is adjusted using APWFM at light loads. Compared to conventional FM, high *Q* is no longer required and the range of switching frequency variation is reduced. The cost and driving loss of the converter system can be low using the proposed control strategy combined with PSR technology because of no need for additional components such as the optocoupler and switches. The experimental results show a general average efficiency improvement of around 4% when the input voltage is below the nominal voltage under light load conditions and around 6% under medium and light load conditions when the input voltage is above or equal to the nominal voltage. High average efficiency under full load conditions is also experimentally verified.

The proposed control strategy is practical and has a high commercial value which can be promoted to other types of switching power supply circuit structures.

## Data Availability

The datasets generated and analysed during the current study are not publicly available due to the nature of this research, participants of this study did not agree for their data to be shared publicly but are available from the corresponding author on reasonable request.
